# Gastrointestinal Tolerance of an Infant Formula Manufactured from Extensively Hydrolysed Protein in Healthy Term Infants

**DOI:** 10.3390/nu15214674

**Published:** 2023-11-04

**Authors:** Lindsey Otten, Elisabeth Schelker, Hanna Petersen, Antonia Nomayo, Romy Conzade, Julia Günther, Andrea Grieger, Frank Jochum, on behalf of the HASI Study Group

**Affiliations:** 1 Department of Pediatrics, Evangelisches Waldkrankenhaus Spandau, Stadtrandstr. 555, 13589 Berlin, Germany; 2 HiPP GmbH & Co. Vertrieb KG, Georg-Hipp-Str. 7, 85276 Pfaffenhofen an der Ilm, Germany; 3 Department of Pediatrics, Faculty of Medicine, Brandenburg Medical School Theodor Fontane (MHB), Fehrbelliner Str. 38, 16816 Neuruppin, Germany

**Keywords:** infant nutrition, infant formula, extensively hydrolysed whey protein, protein hydrolysate, breastfed, gastrointestinal tolerance, digestibility, IGSQ, stool characteristics, sleeping

## Abstract

The evaluation of secondary parameters of a prospective, randomised, controlled, multicentre intervention trial aimed to analyse gastrointestinal tolerance of an infant formula manufactured from extensively hydrolysed whey protein (eHF) compared to intact cow’s milk protein (control formula, CF) in healthy term infants. Infants ≤ 25 days of age, who were exclusively formula-fed, were randomised to receive eHF or CF for at least three months up to 120 days of age. An exclusively breastfed reference group (BF) was included for descriptive comparison. Infants’ gastrointestinal tolerance was evaluated based on stool parameters, the Amsterdam Infant Stool Scale (AISS), the Infant Gastrointestinal Symptom Questionnaire (IGSQ), and sleeping patterns. Of 359 infants included, 297 randomised (eHF: *n* = 149, CF: *n* = 148) and 41 BF infants completed the study per protocol. All tolerance parameters were comparable between eHF and CF. Stool was predominantly soft and yellow in colour. Stool was more frequently green in eHF than CF. BF infants had more frequent stools, which were mainly watery or soft and yellow, and comparable IGSQ scores (descriptive). Irrespective of group, all gastrointestinal and sleep parameters showed signs of maturation with increasing age. In conclusion, eHF showed gastrointestinal tolerance as good as CF in healthy infants. Both formulae were well-tolerated.

## 1. Introduction

Human milk is the gold standard for infant feeding, not only regarding nutritional needs of infants but also for providing bioactive components and ultimately reducing the risk of diseases later in life [1]. Furthermore, the composition of human milk makes it highly digestible and well-tolerated and is considered optimal for infant development. If infants do not receive human milk, infant formula is the most suitable alternative.

Regardless of feeding with human milk or infant formula, a high incidence of gastrointestinal (GI) disturbances is observed in infants [2,3,4]. So-called functional gastrointestinal disorders (FGIDs) are characterised by persistent, recurrent symptoms of the GI tract with no structural or biochemical explanation [5]. The most common FGIDs in infants include infant colic, regurgitation, and functional constipation [5,6]. Based on a review of the literature and the opinions of practicing clinicians, colic, regurgitation, and functional constipation were estimated to occur in 20%, 30%, and 15% in infants up to 12 months, respectively [7]. Of the three FGIDs, the pathophysiology of infant colic appears to be the least understood and may have its causal roots not only in the GI tract, but also the nervous system and/or the psychosocial environment [5]. Although these GI symptoms are typically self-resolving with age, they can have a significant negative impact on the quality of life of the affected infants and their families [8]. While GI problems generally occur in all infants—that is, independent of feeding with human milk or infant formula—some studies have demonstrated an association between formula feeding and the prevalence of FGIDs [9] as well as differences in stool characteristics such as less frequent and harder stools in infants receiving infant formula compared to human milk [10,11,12,13].

Different types of infant formula have been developed in order to meet different needs; each is required to conform to the requirements set out by the European Food Safety Authority (EFSA). Most products are based on cow’s milk protein and are supplemented with nutrients or other bioactive components, such as long-chain unsaturated fatty acids, milk oligosaccharides, and probiotics, in order to best mimic human milk both in nutrient composition and GI tolerance. Studies have suggested that infant formula manufactured from hydrolysed protein may positively affect digestibility and GI symptoms during the first months of life due to the different molecular structures of proteins and peptides [14,15,16].

The purpose of the analysis of the secondary outcome parameters of the “HA Safety in Infants” (HASI) Study was to evaluate the GI tolerance of an infant formula manufactured from extensively hydrolysed whey protein (eHF) compared to an infant formula manufactured from intact protein (control formula, CF) during the first 120 days of life using several well-validated parameters. Exclusively breastfed infants were included for descriptive purposes. Safety and suitability, i.e., weight gain and other growth parameters, of eHF for infant consumption have already been demonstrated within the HASI Study [17,18].

## 2. Methods and Materials

The study design and methods are described here briefly; they have been published in detail elsewhere [18].

### 2.1. Study Design and Population

The study was designed as a prospective, multicentre, randomised, double-blind, parallel-group, controlled, non-inferiority trial. It was carried out from March to October 2021 in 21 centres in four European countries (11 in Bulgaria, one in Germany, eight in Hungary, and one in the Czech Republic). The study was conducted in accordance with ICH Good Clinical Practice and the Declaration of Helsinki as well as with the local legal and regulatory requirements. It was approved by the corresponding ethics committees and registered at ClinicalTrials.gov (NCT04736082).

Healthy term infants from singleton pregnancies whose mothers intended to exclusively feed either formula or human milk were eligible for participation. Further details on inclusion and exclusion criteria have been described elsewhere [18].

Upon written informed consent of both parents or legal guardians (further referred to as “parents” only), eligible formula-fed infants were enrolled into the study and randomised to one of the two infant formula-fed (FF) groups (i.e., eHF or CF). Randomisation was carried out in a double-blinded dynamic manner using country and sex as stratification variables via a centralised randomisation management interface. Breastfed infants were assigned to a non-randomised breastfed reference group (BF).

The presented evaluation of the secondary parameters representing GI tolerance includes the period from enrolment up to 120 days of age. After enrolment (≤25 days of age; V0), infants attended four study visits at 30 ± 3 (V1), 60 ± 3 (V2), 90 ± 3 (V3), and 120 ± 3 (V4) days of age. Exclusive infant formula feeding began no later than 26 days of age and was continued until at least V4.

### 2.2. Study Product

The two infant formulae complied with the requirements defined in Art. 3 Del. Regulation (EU) 2016/127 [19] as well as Art. 5 Directive 2006/141/EC [20]. They had a similar nutrient composition and were isocaloric. The protein content of both infant formulae was 1.9 g/100 kcal. The formulae only differed in the protein structure; one with extensively hydrolysed whey protein (eHF—Peptigen^®^ IF-3080 from Arla Foods Ingredients) and the other with intact cow’s milk protein (CF). Both products contained the probiotic strain *Limosilactobacillus fermentum* CECT5716 (1.0 × 10^7^ CFU/g powdered infant formula at the time of production) and the prebiotic Galactooligosaccharide (GOS) (0.3 g/100 mL). Details on formulae composition have previously been published [18].

### 2.3. Safety Evaluation

In addition and as already published [18], the evaluation of safety was performed throughout the study by documentation of adverse and serious adverse events (AEs, SAEs) coded by the Medical Dictionary for Regulatory Activities (MedDRA) as well as intake of concomitant medications.

### 2.4. Parameters of Gastrointestinal Tolerance

#### 2.4.1. Stool Characteristics

GI tolerance was estimated using data from 3-day diaries completed prior to each visit. From these diaries, stool frequency as average daily number of stools (number of stools/day) and stool characteristics, using the Amsterdam infant stool scale (AISS) [21], were derived. Each collected stool was recorded by parents with respect to the amount in relation to diaper volume (smear, up to 25%, 25–50%, or >50% diaper amount), stool consistency (watery, soft, formed, hard), and stool colour (yellow, orange, green, brown, meconium, or clay-coloured) using the instructions and coloured example images provided in the AISS. The average daily number of stools was calculated as an average from across the three days (or the two available days, missing otherwise). For specific stool characteristics (amount, consistency, or colour), the proportion in each category was calculated based on the number of stools over the three days (or over the two available days, missing otherwise).

#### 2.4.2. Infant Gastrointestinal Symptom Questionnaire (IGSQ)

Furthermore, the IGSQ was completed during each visit to assess GI disturbances [22]. In addition to the 13-item total score from the IGSQ (range 13–65, with lower values indicating better GI tolerance), the following items were assessed individually: frequency of spitting-up (IGSQ, question no. 3), frequency of crying (IGSQ, question no. 7), frequency of crying during or right after feeding (IGSQ, question no. 9), frequency of feeling fussy (IGSQ, question no. 10), and frequency of flatulence (IGSQ, question no. 12).

#### 2.4.3. Sleeping Behaviour

Four adapted questions of the Sleep and Behaviour Questionnaire [23,24] were included in the 3-day diaries to assess the infants’ sleeping behaviour. Based on these questions, the following items were assessed as done by Zandoná et al.: average daily duration of sleep (24 h sleep), average daily duration of sleep during the night (nocturnal sleep), average daily duration of uninterrupted sleep during the night, and average daily time to fall asleep (sleep latency).

### 2.5. Statistical Analysis

#### 2.5.1. Stool Characteristics

Stool characteristics were analysed using a mixed Poisson regression model for repeated measurements, with the visit (from V2 to V4), the infant formula group, the interaction between visit and infant formula group and the sex as fixed effects, and the subject and centre as random effects (with the total number of stools as offset).

#### 2.5.2. IGSQ

The IGSQ total score at each visit was analysed using a linear mixed model for repeated measurements, with the visit (from V2 to V4), the infant formula group, the interaction between visit and infant formula group, and the sex as fixed effects, and the subject and centre as random effects. For the specific analysis of individual questions of the IGSQ at each visit, a mixed logistic ordinal model (for repeated measurements) with the same variables for fixed and random effects as the IGSQ total score analysis was used.

Furthermore, a post-hoc analysis was performed in order to assess any effect of medications or products which may positively influence GI tolerance. In the subgroup of infants who did not receive such products, a mixed linear model (for repeated measurements) was used to compare eHF and CF with regard to IGSQ total score at each visit (the same model as for main statistical analyses). Additionally, to study the differences between the two subgroups (of infants who did or who did not receive medications or products intended to improve GI tolerance), a three-way interaction term with the infant formula group, the visit, and the subgroup was introduced in the previous mixed linear model.

#### 2.5.3. Sleeping Behaviour

Sleeping behaviour was analysed using a linear mixed model (for repeated measurements) with the visit (from V2 to V4), the infant formula group, the interaction between visit and infant formula group, and sex as fixed effects, and the subject and centre as random effects.

#### 2.5.4. Study Population

The data analysis presented herein was performed using the full analysis set (FAS) and per protocol set (PPS). FAS contained all randomised infants in FF and all infants in BF, except those who turned out to be a screening failure, who did not participate in V1 or who did not receive the study product at least once (if in FF). PPS involved infants of FAS without major deviations to the protocol, which included visits outside the visit window, specified medications, or any complementary food and drinks up to V4. The results of GI tolerance are described here for PPS.

The study product was intended to be the only source of nutrition, meaning no breastfeeding, no complementary food, no energy-containing liquids, no feedings of any other infant formula, and no energy-free drinks like water or unsweetened tea were to be consumed until the infant reached the age of 120 days. Infants, who received complementary liquids or foods before 120 days, were not included in the PPS analysis.

The sample size calculation was based on the primary study outcome mean daily weight gain. Details are described elsewhere [18].

BF was not randomised and therefore not included in the inferential statistical analyses. The presented results for BF and comparisons to FF are purely descriptive.

Descriptive analyses were performed by group and by visit for all parameters. Data are presented as mean (SD) unless otherwise indicated.

Inferential tests focused on an explorative two-sided 5% significance level. Statistical analyses were performed using the software SAS^®^ version 9.4 or higher (SAS Institute Inc., Cary, NC, USA).

## 3. Results

### 3.1. Study Participants and Population Characteristics

**Figure 1:**
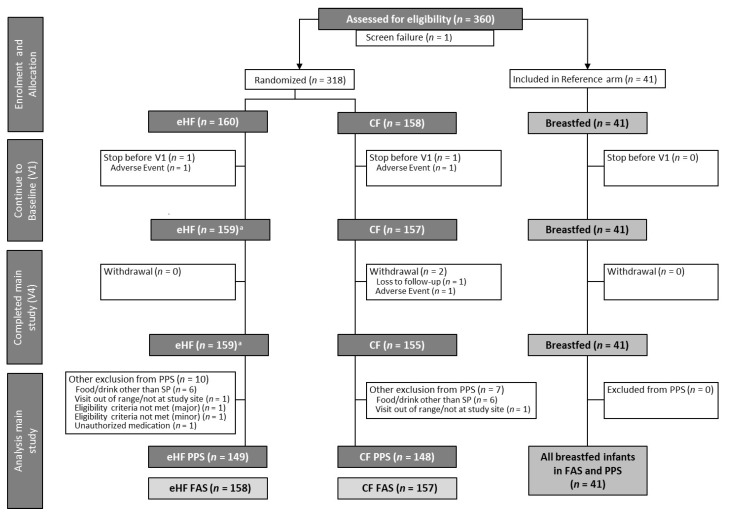
Flow chart of infant enrolment and distribution into formula-fed groups and the breastfed reference group (modified from Otten et al. [18]). ^a^ One infant could not be analysed within FAS due to eligibility criteria not met. V1: 30 ± 3 days; V4: 120 ± 3 days. CF: control formula; eHF: infant formula manufactured from extensively hydrolysed whey protein; FAS: full analysis set; *n*: number of observations; SP: study product; PPS: per protocol set; V: visit.

As reported previously [18], 318 infants were randomised to FF (eHF: *n* = 160, CF: *n* = 158) and 41 enrolled in BF, resulting in a total of 359 enrolled infants (Figure 1).

Birth characteristics, including gestational age, mode of birth, sex, and anthropometric parameters, as well as parental characteristics including maternal age, BMI, and weight gain during pregnancy, were comparable between eHF and CF, as previously reported in detail [18].

Characteristics of BF were similar to FF, except for sex and mode of birth [18]. In BF, there were more females compared to FF (BF: 56.1%, eHF: 43.7%, CF: 43.9%) and the majority of infants were born vaginally (BF: 58.5%, eHF: 41.8%, CF: 41.4%).

### 3.2. Compliance

Study compliance was very high. Seven infants in eHF and nine infants in CF had major deviations in feeding instructions and were thus excluded from PPS; most of these deviations were related to the consumption of low amounts of water and non-sweetened tea. No complementary foods were given before V4. All infants in BF were fully compliant with the feeding instructions, i.e., exclusively breastfed [18].

### 3.3. Safety Evaluation

Details on safety reporting are described in detail elsewhere [18]. In short, comprehensive safety documentation and continuous safety showed no clinically relevant differences regarding the incidence and overall occurrence of adverse events between all groups.

With regard to concomitant medications, approximately 30% of infants received a product which could potentially positively influence GI tolerance, such as simeticone, lactase, or aromatic oils (Table 1).

### 3.4. Gastrointestinal Tolerance

#### 3.4.1. Stool Characteristics

**Figure 2:**
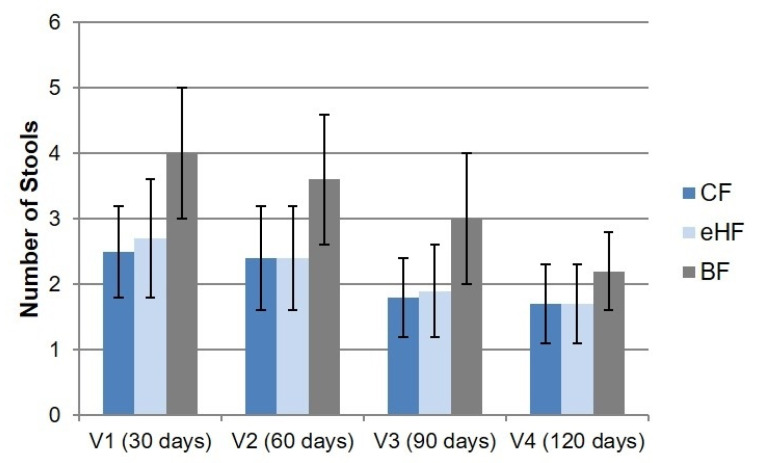
Mean daily stool frequency (daily number of stools; mean ± SD) from 3-day diary (PPS). Missing values in eHF: V4: *n* = 1. BF: breastfed reference group; CF: control formula; eHF: infant formula manufactured from extensively hydrolysed whey protein; *n*: number of observations; PPS: per protocol set; SD: standard deviation; V: visit.

Daily stool frequency varied strongly among individuals but did not differ between FF groups at any visit (Figure 2). Daily stool frequency decreased from V1 to V4 in both FF groups from mean 2.7 ± 0.9 (range: 0.0–6.0) and 2.5 ± 0.7 (range: 0.7–4.7) stools per day at V1 in eHF and CF, respectively, to 1.7 ± 0.6 (range: eHF: 0.7–4.3; CF: 0.7–4.0) stools per day at V4 in both FF groups.

While mean daily stool frequency in BF also decreased from V1 to V4, trending towards the levels of FF infants, it was higher at all visits compared to FF (4.0 ± 1.0 (range: 1.7–6.3) stools per day at V1 to 2.2 ± 0.6 (range: 1.3–4.3) stools per day at V4) (Figure 2).

Although stool amount varied over time, no difference was observed between FF groups at any visit (Figure S1). The most prevalent stool amount of FF infants was 25–50% of diaper volume, and FF generally reported a higher amount of stool compared to BF up to V3. This difference became less apparent at V4, as stool amount per diaper increased over time in BF, nearing the amounts observed in FF groups. While approximately 50% of FF infants at V1, V2, and V3, and 65% at V4 reported a stool amount of 25–50% of diaper volume, this amount was reported for approximately 30% of BF infants at V1, 50% at V2, 40% at V3, and 75% at V4 (Figure S1).

**Figure 3:**
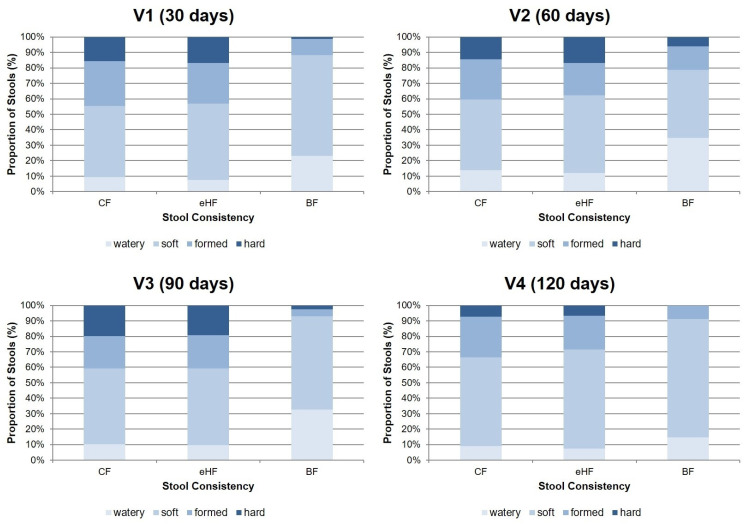
Stool consistency from 3-day diary (% ± SD; PPS). Missing values in eHF: V1: *n* = 2; V4: *n* = 1. Missing values in CF: V3: *n* = 1. BF: breastfed reference group; CF: control formula; eHF: infant formula manufactured from extensively hydrolysed whey protein; PPS: per protocol set; V: visit.

Regarding stool consistency, no differences were observed between FF groups at any visit (Figure 3). The predominant proportion of stools in FF at all visits was categorised as soft (ranging from 45.8 ± 36.9% of stools in CF at V2 to 64.1 ± 33.4% in eHF at V4), followed by formed stool (ranging from 21.0 ± 27.9% of stools at V2 in eHF to 28.8 ± 30.1% at V1 in CF). There was a low overall rate of watery and hard stools (<20% of stools reported in FF throughout all visits) and (near) absence of constipation or diarrhoea in both FF groups [18].

Soft stool was also the predominant consistency in BF at all visits (ranging from 43.9 ± 22.6% of stools at V2 to 76.5 ± 19.8% at V4), followed by watery stool (ranging from 14.7 ± 15.9% of stools at V4 to 34.7 ± 22.0% at V2). In a descriptive comparison, BF had a higher prevalence of watery stools than FF and lower prevalence of formed and hard stools throughout the study period.

**Figure 4:**
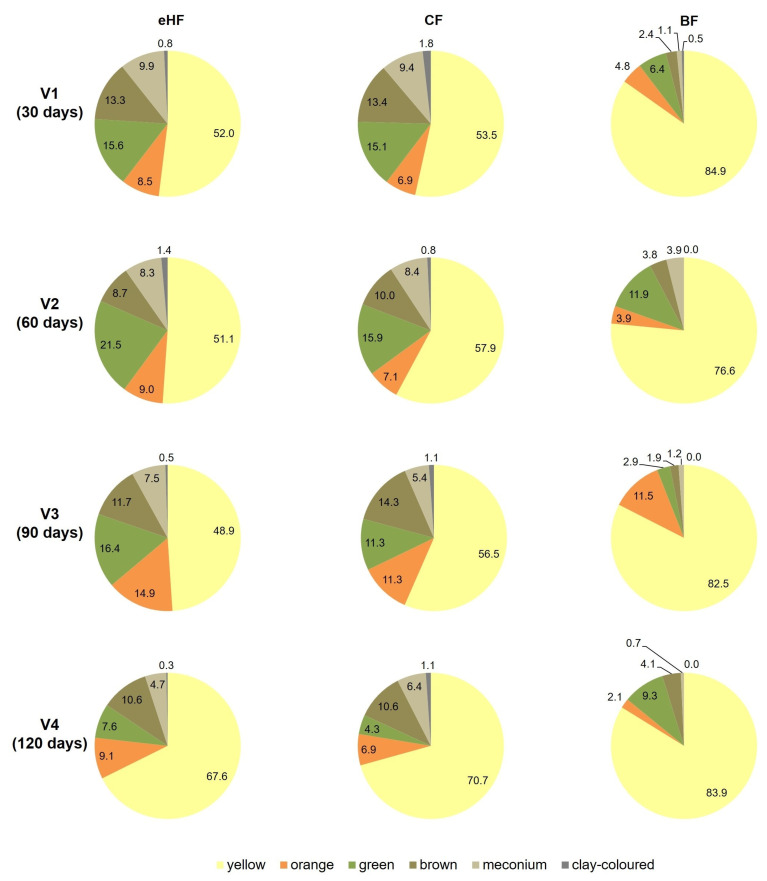
Stool colour from 3-day diary (%; PPS). Missing values in eHF: V1: *n* = 2; V4: *n* = 1. Missing values in CF: V3: *n* = 1. BF: breastfed reference group; CF: control formula; eHF: infant formula manufactured from extensively hydrolysed whey protein; PPS: per protocol set; V: visit.

Stool colour was largely comparable between FF groups, except for green stools, which were more common in eHF than CF (ranging from 7.6 ± 18.9% at V4 to 21.5 ± 26.4% at V2 in eHF and 4.3 ± 12.0% at V4 to 15.9 ± 19.9% at V2 in CF). This difference in stool colour was not significant in the PPS at any visit but reached significance at V3 only in the FAS (V3: eHF 16.9 ± 25.8% vs. CF 10.8 ± 16.1% stools with green colour over 3 days, *p* = 0.0385). Throughout the study period, the majority of stools in both groups were yellow (Figure 4).

Also in BF, the majority (>75%) of stools were yellow throughout the study period (V1: 84.9 ± 17.7%; V4: 83.9 ± 20.9%). Each of the other colours was observed in less than 12% of the stools reported at each visit (Figure 4).

#### 3.4.2. IGSQ

Mean IGSQ total score decreased from V1 to V4 in both FF groups and did not differ between FF groups at any visit (Table 2).

Similarly, in BF, mean IGSQ score decreased throughout the study period and showed a tendency to lower values compared to FF until V3. At V4, this trend was no longer visible (Table 2).

Consistent with IGSQ total scores, selected individual question scores did not differ between FF groups (Table S1) and decreased with age in FF and BF groups.

The safety evaluation revealed that about one third of the infants preventively received products with a possible positive impact on GI tolerance (Table 1). In the post-hoc analysis, there was no difference in IGSQ total score between eHF and CF at any visit in the subgroup of infants who did not receive such products (Table S2). Furthermore, no differences were observed when including the three-way interaction term with the subgroup information (Table S3).

#### 3.4.3. Sleeping Behaviour

There were no differences observed in any of the sleep parameters (24 h sleep, nocturnal sleep, interrupted nocturnal sleep, time to fall asleep) between eHF and CF throughout the study period. While 24 h sleep decreased from V1 to V4 by approximately 18% in both eHF and CF, the duration of nocturnal sleep increased in both groups by about 9% in eHF and 10% in CF (Table 3). The same dynamic was observed in BF (Table 3).

The mean duration of uninterrupted nocturnal sleep increased across all groups from approximately 4.0 ± 1.8 h per night at V1 to 6.2 ± 1.1 h per night at V4, while the time to fall asleep decreased from approximately 0.8 ± 0.4 h per day at V1 to a mean of 0.5 ± 0.2 h per day at V4 in all groups (Table S4).

## 4. Discussion

In this secondary analysis of a multicentre, randomised, controlled, double-blind trial, the GI tolerance of eHF was compared to that of CF. No significant differences were observed during the first 120 days of life, as reflected by similar stool characteristics, IGSQ and sleeping patterns.

The daily number of stools decreased over time, with no significant differences between FF groups as previously shown elsewhere [13]. The high frequency of defecation in early life is thought to be related to the not-yet-fully-matured gut [25]. The age-related decline in the frequency of stools points toward a maturation of the water-conserving capacity of the gut [25].

The most commonly reported stool consistency of FF infants was soft, which is in line with previous findings with infant formulae manufactured from hydrolysed or intact protein containing pre- and/or probiotics [10,13,26]. The prebiotic GOS and the probiotic *L. fermentum* were present in both study formulae. GOS has been shown to stimulate colonisation of intestinal Bifidobacteria and Lactobacilli [26] and to produce softer stools [27,28]. More recently, soft stools were predominantly observed in infants consuming formulae with intact protein in combination with GOS and *L. fermentum* [29]. In our study, there was a low prevalence of hard and watery stools, supporting the good GI tolerability of both products.

Overall, stool colour was comparable between FF groups. A yellow stool colour was dominant, but orange, brown, green, meconium, and clay-coloured stools were also noted. This wide variety of stool colours is common in healthy infants [30]. Since the formulae differed only in their source of protein, the slightly higher prevalence of green stools reported throughout the study in eHF is most likely attributable to the difference in protein structure (hydrolysed versus intact protein). The occurrence of green stools is commonly seen in infants consuming infant formulae manufactured from hydrolysed protein [31,32] as these are absorbed and metabolised differently than intact proteins. Generally, green stools in FF infants are considered normal [33].

Throughout the study period, median IGSQ scores were highly comparable between FF groups and were below proposed norm-reference values [34], suggesting that both study products are well-tolerated. The IGSQ total scores decreased over time, indicating increasing GI maturity [34]. Median IGSQ norm-reference values of 28 and 27 were suggested for infants at the ages of 0–2 months and 2–4 months, respectively, for both FF and BF infants [34]. Pados et al. reported that the median IGSQ score was reduced by four points over four months in infants from the age of 0–2 months to 4–6 months [34]. In our study, we observed a reduction in median IGSQ score of up to ten points in only three months. Consistent with IGSQ total scores, individual question scores reflecting infant GI symptoms in the previous week (frequency of spitting up, crying during or right after feeding, fussiness, flatulence) also decreased with age in all groups, which is in accordance with findings by Tunc et al. showing that GI symptoms decrease with age as the GI system matures [25].

Furthermore, there were no differences in sleeping characteristics between FF groups, with decreasing 24 h sleep and fewer sleep interruptions during the night as age increased. Rivkees et al. described a progressive maturation of the circadian system in infants [35], which seems to be in line with the GI maturation with age in our study and supports a good GI tolerance of both formulae. Moreover, infants in FF groups received the same numbers of feedings as well as infant formula volume per day, which may have contributed to the similar sleeping patterns [18].

No statistical comparison to the non-randomised BF reference group was performed; thus, the following discussion is based on descriptive statistics only. In accordance with previous studies, exclusively breastfed infants have more frequent and softer stools than exclusively formula-fed infants [10,11,12,13]. One reason for softer stools may be a higher content and wider variety of oligosaccharide types present in human milk (human milk oligosaccharides, (HMO)) [36,37], ranging between 0.5 and 1.5 g/100 mL. Although both formulae in the current study did include oligosaccharides, these did not reach the complexity and levels of human milk.

In the present study, GI tolerance assessed by the IGSQ questionnaire was comparable between the BF and FF groups, which may have contributed to the similar feeding amounts between these two groups, as already published [18]. Furthermore, the good tolerance of both formulae and comparable feeding frequencies may have contributed to the similar sleeping behaviour seen in the FF and BF groups. Whether BF or FF infants sleep longer or have longer uninterrupted sleep phases is inconsistent in the literature [38,39,40].

Notably, in the current study, approximately one third of all infants (FF and BF) received a medication or preparation, usually preventatively, aiming to improve GI tolerance and thereby potentially preventing some symptoms of intolerance that may have otherwise occurred in the study cohort. For this reason, post-hoc statistical analyses were performed. These revealed no differences in IGSQ total score between eHF and CF in the subgroup of infants who did not receive such products. According to these results, it can be assumed that the intake of the recorded products intended to improve GI tolerance had no measurable effect in this cohort. Even so, the relatively prevalent intake of products intended to improve GI tolerance is a limitation in this study.

Furthermore, due to the nature of the trial design as a safety study, no data on infant microbiome are available, which may have provided further insight into GI tolerance.

The presented evaluation of secondary outcome parameters is further limited in that the sample size calculation was based on the primary objective. Nevertheless, the high compliance and low dropout rate in this study together with the use of several validated tools resulted in comprehensive data on GI tolerance, stool characteristics, and sleeping patterns at multiple time points. Furthermore, the inclusion of a reference breastfed group allowed the descriptive comparison of infant formula tolerance to human milk as the ideal and recommended nutrient source for infants.

## 5. Conclusions

The infant formula manufactured from extensively hydrolysed whey protein used in this study led to comparable results in various parameters of GI tolerance, including stool characteristics, IGSQ, and sleep behaviour, as the control infant formula manufactured from intact cow’s milk protein. Based on these findings, it can be concluded that the extensively hydrolysed whey protein-based infant formula is equally well-tolerated as the control infant formula in exclusively formula-fed infants during the first 4 months of life. Both formulae showed good GI tolerance. Nevertheless, future research should continue the effort in developing infant formula that results in stool consistency and GI symptoms in early infancy closer to those resulting from exclusive breastfeeding.

## Figures and Tables

**Table 1:** Receipt of products which may positively influence GI tolerance (PPS).

Products Intended to Positively Influence GI Tolerance	All Infants (*n* = 338)	eHF (*n* = 149)	CF (*n* = 148)	BF (*n* = 41)
Yes	92 (27.2)	40 (26.8)	43 (29.1)	9 (22.0)
No	246 (72.8)	109 (73.2)	105 (70.9)	32 (78.0)

Values presented as *n* (%). BF: breastfed reference group; CF: control formula; eHF: infant formula manufactured from extensively hydrolysed whey protein; GI: gastrointestinal; *n*: number of observations; PPS: per protocol set.

**Table 2:** IGSQ 13-item total score (a.u., range 13–65) from 3-day diary (PPS).

Visit	Age at Visit (Days)		eHF (*n* = 149)	CF (*n* = 148)	BF (*n* = 41)
1	30	Mean (SD)	26.5 (7.2)	26.5 (6.8)	24.8 (6.5)
		Median (Q1; Q3)	26.0 (20.2; 31.4)	27.1 (20.2; 31.2)	24.6 (18.9; 27.4)
2	60	Mean (SD)	24.0 (6.3)	23.3 (6.7)	22.4 (6.1)
		Median (Q1; Q3)	23.0 (19.5; 28.9)	22.8 (18.0; 27.3)	21.7 (17.9; 25.0)
3	90	Mean (SD)	22.1 (4.9)	21.8 (4.8)	21.1 (4.5)
		Median (Q1; Q3)	21.7 (18.2; 24.8)	21.3 (18.8; 24.9)	21.7 (18.9; 23.8)
4	120	Mean (SD)	18.5 (4.2)	18.4 (4.4)	19.1 (4.6)
		Median (Q1; Q3)	17.3 (15.9; 22.1)	17.3 (14.6; 21.9)	20.1 (14.6; 22.1)

Missing values in eHF: V4: *n* = 1. Missing values in CF: V2: *n* = 1. a.u.: arbitrary units; BF: breastfed reference group; CF: control formula; eHF: infant formula manufactured from extensively hydrolysed whey protein; IGSQ: Infant Gastrointestinal Symptom Questionnaire; *n*: number of observations; max: maximum; min: minimum; PPS: per protocol set; SD: standard deviation; Q: Quartile.

**Table 3:** Mean 24 h sleep (hours/day) and duration of nocturnal sleep (hours/day) from 3-day diary (PPS).

Visit	Age at Visit (Days)	24 h Sleep			Nocturnal Sleep		
		eHF (*n* = 149)	CF (*n* = 148)	BF (*n* = 41)	eHF (*n* = 149)	CF (*n* = 148)	BF (*n* = 41)
1	30	16.3 (1.9)	16.3 (1.8)	16.0 (2.0)	7.6 (1.7)	7.7 (1.9)	7.7 (2.3)
		(11.3; 22.3)	(11.0; 23.0)	(12.9; 19.7)	(4.7; 11.7)	(2.3; 12.0)	(4.8; 12.0)
2	60	15.1 (2.3)	15.0 (2.2)	14.9 (2.1)	7.6 (1.5)	7.7 (1.6)	7.6 (1.6)
		(9.3; 22.0)	(10.6; 22.7)	(12.1; 21.7)	(5.0; 12.0)	(4.3; 11.7)	(5.1; 10.5)
3	90	13.8 (2.8)	13.8 (2.5)	13.1 (2.4)	7.7 (1.5)	7.8 (1.6)	7.7 (1.5)
		(9.8; 22.2)	(9.9; 22.5)	(10.6; 21.3)	(4.7; 12.3)	(5.1; 12.0)	(5.7; 11.2)
4	120	13.3 (2.7)	13.3 (2.5)	13.2 (2.8)	8.3 (1.0)	8.5 (1.2)	8.6 (1.0)
		(8.0; 21.2)	(8.9; 22.3)	(9.7; 20.0)	(4.7; 11.1)	(6.0; 11.7)	(7.0; 10.8)

Values presented as mean (SD), (min; max). BF: breastfed reference group; CF: control formula; eHF: infant formula manufactured from extensively hydrolysed whey protein; *n*: number of observations; PPS: per protocol set; SD: standard deviation.

## Data Availability

The original contributions presented in the study are included in the article/Supplementary Material. Further inquiries can be directed to the corresponding author/s.

## Supplementary Materials

The following supporting information can be downloaded at https://www.mdpi.com/article/10.3390/nu15214674/s1, Figure S1: Proportion of stool amount (in relation to diaper volume) per diaper from 3-day diary (PPS); Table S1: Selected individual items from the IGSQ (PPS); Table S2: Comparison of IGSQ 13-item total score (a.u., range 13–65) by visit in the subgroup of infants who did not take GI tolerance medications (linear mixed model for repeated measurements) (PPS); Table S3: Comparison of IGSQ 13-item total score (a.u., range 13–65) between the two subgroups of infants who did or did not receive GI tolerance medication (three-way interaction term with infant formula group, visit, and subgroup in linear mixed model for repeated measurements) (PPS); Table S4: Mean duration of uninterrupted nocturnal sleep (hours/night) and mean daily time to fall asleep (hours/day) from 3-day diary (PPS).
