# Supplementary material for: Gastrointestinal Tolerance of an Infant Formula Manufactured from Extensively Hydrolysed Protein in Healthy Term Infants
Source: Nutrients. 2023 Nov 4;15(21):4674. doi: 10.3390/nu15214674 (PMC10647512; doi:10.3390/nu15214674)
Supplement: Supplementary file 1 [file nutrients-15-04674-s001.zip › nutrients-2672467-supplementary.pdf]

**Supplementary Materials** (Otten et al. Nutrients 2023)

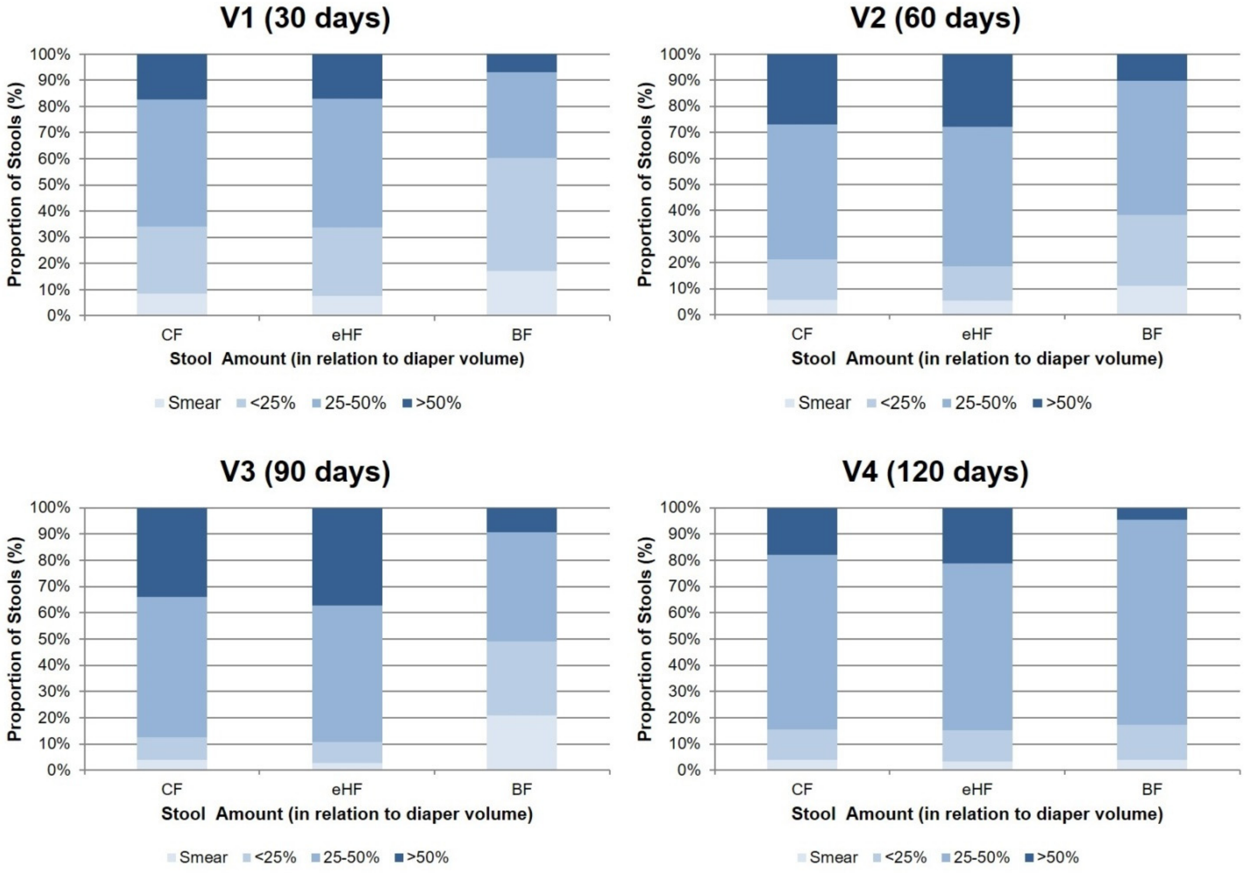


Figure S1. Proportion of stool amount (in relation to diaper volume) per diaper from 3-day diary (PPS).

Missing values in eHF: V1: *n* = 2; V4: *n* = 1.

Missing values in CF: V3: *n* = 1.

BF: breastfed reference group; CF: control formula; eHF: infant formula manufactured from extensively hydrolysed whey protein; PPS: per protocol set; V: visit.

Table S1. Selected individual items from the IGSQ (PPS)

| Visit | Age at Visit (Days) | | eHF (*n* = 149) | CF (*n* = 148) | BF (*n* = 41) |
|---|---|---|---|---|---|
| No. 3: Frequency of spitting up: "Thinking about a usual day in the past week, how many times did milk come out of your baby's mouth?" | | | | | |
| 1 | 30 | 0 times in a usual day | 77 (51.7) | 79 (53.4) | 21 (51.2) |
| | | 1 time in a usual day | 35 (23.5) | 36 (24.3) | 11 (26.8) |
| | | 2 to 3 times in a usual day | 32 (21.5) | 31 (20.9) | 7 (17.1) |
| | | 4 to 6 times in a usual day | 4 (2.7) | 2 (1.4) | 2 (4.9) |
| | | 7 or more times in a usual day | 0 (0.0) | 0 (0.0) | 0 (0.0) |
| | | Do not know or no response | 1 (0.7) | 0 (0.0) | 0 (0.0) |
| 2 | 60 | 0 times in a usual day | 83 (55.7) | 85 (57.4) | 22 (53.7) |
| | | 1 time in a usual day | 44 (29.5) | 44 (29.7) | 14 (34.1) |
| | | 2 to 3 times in a usual day | 19 (12.8) | 19 (12.8) | 5 (12.2) |
| | | 4 to 6 times in a usual day | 3 (2.0) | 0 (0.0) | 0 (0.0) |
| | | 7 or more times in a usual day | 0 (0.0) | 0 (0.0) | 0 (0.0) |
| | | Do not know or no response | 0 (0.0) | 0 (0.0) | 0 (0.0) |
| 3 | 90 | 0 times in a usual day | 91 (61.1) | 82 (55.4) | 17 (41.5) |
| | | 1 time in a usual day | 32 (21.5) | 41 (27.7) | 19 (46.3) |
| | | 2 to 3 times in a usual day | 23 (15.4) | 24 (16.2) | 4 (9.8) |
| | | 4 to 6 times in a usual day | 3 (2.0) | 1 (0.7) | 0 (0.0) |
| | | 7 or more times in a usual day | 0 (0.0) | 0 (0.0) | 0 (0.0) |
| | | Do not know or no response | 0 (0.0) | 0 (0.0) | 1 (2.4) |
| 4 | 120 | 0 times in a usual day | 113 (75.8) | 117 (79.1) | 27 (65.9) |
| | | 1 time in a usual day | 30 (20.1) | 25 (16.9) | 12 (29.3) |
| | | 2 to 3 times in a usual day | 4 (2.7) | 6 (4.1) | 2 (4.9) |
| | | 4 to 6 times in a usual day | 1 (0.7) | 0 (0.0) | 0 (0.0) |
| | | 7 or more times in a usual day | 0 (0.0) | 0 (0.0) | 0 (0.0) |
| | | Do not know or no response | 1 (0.7) | 0 (0.0) | 0 (0.0) |
| No. 7: Frequency of crying: „Thinking about the past week, how much total time did your baby usually cry in a day?" | | | | | |
| 1 | 30 | Less than 10 minutes in a day | 29 (19.5) | 27 (18.2) | 10 (24.4) |
| | | 10 to 30 minutes in a day | 52 (34.9) | 60 (40.5) | 11 (26.8) |
| | | 30 minutes to 1 hour in a day | 39 (26.2) | 33 (22.3) | 8 (19.5) |
| | | 1 to 2 hours in a day | 22 (14.8) | 19 (12.8) | 7 (17.1) |
| | | 2 or more hours in a day | 3 (2.0) | 6 (4.1) | 3 (7.3) |
| | | Do not know or no response | 4 (2.7) | 3 (2.0) | 2 (4.9) |
| 2 | 60 | Less than 10 minutes in a day | 37 (24.8) | 45 (30.4) | 14 (34.1) |
| | | 10 to 30 minutes in a day | 42 (28.2) | 47 (31.8) | 11 (26.8) |
| | | 30 minutes to 1 hour in a day | 50 (33.6) | 37 (25.0) | 13 (31.7) |
| | | 1 to 2 hours in a day | 17 (11.4) | 17 (11.5) | 2 (4.9) |
| | | 2 or more hours in a day | 2 (1.3) | 1 (0.7) | 0 (0.0) |
| | | Do not know or no response | 1 (0.7) | 1 (0.7) | 1 (2.4) |
| 3 | 90 | Less than 10 minutes in a day | 49 (32.9) | 48 (32.4) | 14 (34.1) |
| | | 10 to 30 minutes in a day | 43 (28.9) | 46 (31.1) | 16 (39.0) |
| | | 30 minutes to 1 hour in a day | 38 (25.5) | 38 (25.7) | 11 (26.8) |
| | | 1 to 2 hours in a day | 19 (12.8) | 15 (10.1) | 0 (0.0) |
| | | 2 or more hours in a day | 0 (0.0) | 0 (0.0) | 0 (0.0) |
| | | Do not know or no response | 0 (0.0) | 1 (0.7) | 0 (0.0) |

| Visit | Age at Visit (Days) | | eHF (*n* = 149) | CF (*n* = 148) | BF (*n* = 41) |
|---|---|---|---|---|---|
| 4 | 120 | Less than 10 minutes in a day | 68 (45.6) | 72 (48.6) | 21 (51.2) |
| | | 10 to 30 minutes in a day | 38 (25.5) | 41 (27.7) | 5 (12.2) |
| | | 30 minutes to 1 hour in a day | 31 (20.8) | 22 (14.9) | 9 (22.0) |
| | | 1 to 2 hours in a day | 11 (7.4) | 12 (8.1) | 5 (12.2) |
| | | 2 or more hours in a day | 0 (0.0) | 0 (0.0) | 0 (0.0) |
| | | Do not know or no response | 1 (0.7) | 1 (0.7) | 1 (2.4) |
| No. 9: Frequency of crying during or right after feeding: “Thinking about the past week, how many times did your baby cry during or right after a feeding because the milk bothered your baby?” | | | | | |
| 1 | 30 | 0 times in the week | 88 (59.1) | 89 (60.1) | 21 (51.2) |
| | | 1 time in the week | 39 (26.2) | 40 (27.0) | 13 (31.7) |
| | | 2 times in the week | 14 (9.4) | 10 (6.8) | 3 (7.3) |
| | | 3 times in the week | 2 (1.3) | 4 (2.7) | 1 (2.4) |
| | | 4 or more times in the week | 0 (0.0) | 1 (0.7) | 0 (0.0) |
| | | Do not know or no response | 6 (4.0) | 4 (2.7) | 3 (7.3) |
| 2 | 60 | 0 times in the week | 83 (55.7) | 89 (60.1) | 22 (53.7) |
| | | 1 time in the week | 40 (26.8) | 34 (23.0) | 11 (26.8) |
| | | 2 times in the week | 21 (14.1) | 20 (13.5) | 7 (17.1) |
| | | 3 times in the week | 3 (2.0) | 4 (2.7) | 1 (2.4) |
| | | 4 or more times in the week | 1 (0.7) | 0 (0.0) | 0 (0.0) |
| | | Do not know or no response | 1 (0.7) | 1 (0.7) | 0 (0.0) |
| 3 | 90 | 0 times in the week | 93 (62.4) | 93 (62.8) | 19 (46.3) |
| | | 1 time in the week | 43 (28.9) | 42 (28.4) | 11 (26.8) |
| | | 2 times in the week | 10 (6.7) | 11 (7.4) | 11 (26.8) |
| | | 3 times in the week | 2 (1.3) | 2 (1.4) | 0 (0.0) |
| | | 4 or more times in the week | 0 (0.0) | 0 (0.0) | 0 (0.0) |
| | | Do not know or no response | 1 (0.7) | 0 (0.0) | 0 (0.0) |
| 4 | 120 | 0 times in the week | 104 (69.8) | 105 (70.9) | 18 (43.9) |
| | | 1 time in the week | 40 (26.8) | 32 (21.6) | 17 (41.5) |
| | | 2 times in the week | 2 (1.3) | 9 (6.1) | 3 (7.3) |
| | | 3 times in the week | 1 (0.7) | 0 (0.0) | 1 (2.4) |
| | | 4 or more times in the week | 0 (0.0) | 1 (0.7) | 0 (0.0) |
| | | Do not know or no response | 2 (1.3) | 1 (0.7) | 2 (4.9) |
| No. 10: Frequency of feeling fussy: “Thinking about the past week, on how many days was your baby fussy?” | | | | | |
| 1 | 30 | 0 day | 82 (55.0) | 85 (57.4) | 27 (65.9) |
| | | 1 day | 26 (17.4) | 22 (14.9) | 7 (17.1) |
| | | 2 or 3 days | 39 (26.2) | 38 (25.7) | 7 (17.1) |
| | | 4 or 5 days | 2 (1.3) | 3 (2.0) | 0 (0.0) |
| | | 6 or more days | 0 (0.0) | 0 (0.0) | 0 (0.0) |
| 2 | 60 | 0 day | 92 (61.7) | 99 (66.9) | 26 (63.4) |
| | | 1 day | 20 (13.4) | 16 (10.8) | 7 (17.1) |
| | | 2 or 3 days | 37 (24.8) | 33 (22.3) | 8 (19.5) |
| | | 4 or 5 days | 0 (0.0) | 0 (0.0) | 0 (0.0) |
| | | 6 or more days | 0 (0.0) | 0 (0.0) | 0 (0.0) |
| 3 | 90 | 0 day | 101 (67.8) | 105 (70.9) | 24 (58.5) |
| | | 1 day | 30 (20.1) | 27 (18.2) | 8 (19.5) |
| | | 2 or 3 days | 18 (12.1) | 16 (10.8) | 9 (22.0) |
| | | 4 or 5 days | 0 (0.0) | 0 (0.0) | 0 (0.0) |
| | | 6 or more days | 0 (0.0) | 0 (0.0) | 0 (0.0) |

| Visit | Age at Visit (Days) | | eHF (*n* = 149) | CF (*n* = 148) | BF (*n* = 41) |
|---|---|---|---|---|---|
| 4 | 120 | 0 day | 114 (76.5) | 115 (77.7) | 24 (58.5) |
| | | 1 day | 21 (14.1) | 14 (9.5) | 13 (31.7) |
| | | 2 or 3 days | 14 (9.4) | 19 (12.8) | 4 (9.8) |
| | | 4 or 5 days | 0 (0.0) | 0 (0.0) | 0 (0.0) |
| | | 6 or more days | 0 (0.0) | 0 (0.0) | 0 (0.0) |
| No. 12: Frequency of flatulence: "Thinking about the past week, how many times in a usual day was your baby gassy?" | | | | | |
| 1 | 30 | 0 times in a usual day | 14 (9.4) | 22 (14.9) | 3 (7.3) |
| | | 1 time in a usual day | 40 (26.8) | 47 (31.8) | 20 (48.8) |
| | | 2 to 3 times in a usual day | 63 (42.3) | 52 (35.1) | 16 (39.0) |
| | | 4 to 6 times in a usual day | 25 (16.8) | 20 (13.5) | 1 (2.4) |
| | | 7 or more times in a usual day | 4 (2.7) | 5 (3.4) | 0 (0.0) |
| | | Do not know or no response | 3 (2.0) | 2 (1.4) | 1 (2.4) |
| 2 | 60 | 0 times in a usual day | 33 (22.1) | 35 (23.6) | 13 (31.7) |
| | | 1 time in a usual day | 50 (33.6) | 64 (43.2) | 17 (41.5) |
| | | 2 to 3 times in a usual day | 48 (32.2) | 36 (24.3) | 11 (26.8) |
| | | 4 to 6 times in a usual day | 14 (9.4) | 8 (5.4) | 0 (0.0) |
| | | 7 or more times in a usual day | 4 (2.7) | 4 (2.7) | 0 (0.0) |
| | | Do not know or no response | 0 (0.0) | 1 (0.7) | 0 (0.0) |
| 3 | 90 | 0 times in a usual day | 56 (37.6) | 58 (39.2) | 21 (51.2) |
| | | 1 time in a usual day | 55 (36.9) | 60 (40.5) | 19 (46.3) |
| | | 2 to 3 times in a usual day | 27 (18.1) | 23 (15.5) | 1 (2.4) |
| | | 4 to 6 times in a usual day | 9 (6.0) | 7 (4.7) | 0 (0.0) |
| | | 7 or more times in a usual day | 2 (1.3) | 0 (0.0) | 0 (0.0) |
| | | Do not know or no response | 0 (0.0) | 0 (0.0) | 0 (0.0) |
| 4 | 120 | 0 times in a usual day | 89 (59.7) | 90 (60.8) | 24 (58.5) |
| | | 1 time in a usual day | 31 (20.8) | 33 (22.3) | 16 (39.0) |
| | | 2 to 3 times in a usual day | 19 (12.8) | 15 (10.1) | 1 (2.4) |
| | | 4 to 6 times in a usual day | 8 (5.4) | 7 (4.7) | 0 (0.0) |
| | | 7 or more times in a usual day | 1 (0.7) | 1 (0.7) | 0 (0.0) |
| | | Do not know or no response | 1 (0.7) | 2 (1.4) | 0 (0.0) |

Values presented as *n* (%). No missing values.

The statistical analysis with a logistic ordinal mixed model for repeated measurements showed a significant effect of visit on all individual questions ($p<0.001$).

BF: breastfed group; CF: control formula; eHF: infant formula manufactured from extensively hydrolysed whey protein; *n*: number of observations; No.: number; PPS: per protocol set

Table S2. Comparison of IGSQ 13-item total score (a.u., range 13-65) by visit in the subgroup of infants who did not take GI tolerance medications (linear mixed model for repeated measurements) (PPS)

| Visit | Group | N | LSM | SEM | 95% confidence interval (2-sided) | Two-sided superiority p value |
|---|---|---|---|---|---|---|
| 2 | eHF | 109 | 22.62 | 1.0169 | [20.51;24.73] | |
| | CF | 105 | 22.02 | 1.0304 | [19.89;24.15] | |
| | eHF vs CF | | 0.60 | 0.8198 | [-1.02;2.21] | 0.4679 |
| 3 | eHF | 109 | 21.07 | 0.9315 | [19.09;23.05] | |
| | CF | 105 | 20.67 | 0.9429 | [18.66;22.67] | |
| | eHF vs CF | | 0.40 | 0.5770 | [-0.73;1.54] | 0.4843 |
| 4 | eHF | 108 | 17.46 | 0.9465 | [15.46;19.46] | |
| | CF | 105 | 17.76 | 0.9575 | [15.74;19.78] | |
| | eHF vs CF | | -0.30 | 0.6241 | [-1.53;0.93] | 0.6326 |
| | Product | | | | | 0.5683 |
| | Visit | | | | | <0.0001 |
| | Sex | | | | | 0.5818 |
| | Product*Visit | | | | | 0.6166 |

Missing values in eHF: V4: *n* = 1; V2: *n* = 1.

a.u.: arbitrary units; BF: breastfed group; CF: control formula; eHF: infant formula manufactured from extensively hydrolysed whey protein; GI: gastrointestinal; IGSQ: Infant Gastrointestinal Symptoms Questionnaire; LSM: least square means; N: number of observations; PPS: per protocol set; SEM: standard error of mean

Table S3. Comparison of IGSQ 13-item total score (a.u., range 13-65) between the two subgroups of infants who did or did not receive GI tolerance medication (three-way interaction term with infant formula group, visit, and subgroup in linear mixed model for repeated measurements) (PPS)

| Visit | GI tolerance medication | Group | N | LSM | SEM | 95% confidence interval (2-sided) | Two-sided superiority p value |
|---|---|---|---|---|---|---|---|
| 2 | Yes | eHF | 149 | 23.56 | 1.2803 | [20.99;26.12] | |
| | | CF | 147 | 23.34 | 1.2623 | [20.81;25.87] | |
| | No | eHF | 149 | 22.71 | 0.9958 | [20.64;24.77] | |
| | | CF | 147 | 22.10 | 1.0091 | [20.01;24.19] | |
| 3 | Yes | eHF | 149 | 20.79 | 1.1221 | [18.51;23.07] | |
| | | CF | 148 | 21.24 | 1.1042 | [18.99;23.49] | |
| | No | eHF | 149 | 21.16 | 0.9230 | [19.21;23.12] | |
| | | CF | 148 | 20.75 | 0.9346 | [18.77;22.72] | |
| 4 | Yes | eHF | 148 | 17.14 | 1.1037 | [14.89;19.39] | |
| | | CF | 148 | 16.68 | 1.0868 | [14.46;18.90] | |
| | No | eHF | 148 | 17.56 | 0.9155 | [15.61;19.50] | |
| | | CF | 148 | 17.84 | 0.9263 | [15.88;19.80] | |
| | | Product | | | | | 0.6725 |
| | | Visit | | | | | <0.0001 |
| | | Sex | | | | | 0.7713 |
| | | GI tolerance medication | | | | | 0.8097 |
| | | Product*Visit | | | | | 0.8965 |
| | | Visit*GI tolerance medication | | | | | 0.1726 |
| | | Product*GI tolerance medication | | | | | 0.8222 |
| | | Product*Visit*GI tolerance medication | | | | | 0.5689 |

Missing values in subgroup of infants who did not take GI tolerance medications: in eHF: V4: *n* = 1; V2: *n* = 1. Missing values in subgroup infants who did take GI tolerance medications: in CF: V2, *n*=1.

a.u.: arbitrary units; BF: breastfed group; CF: control formula; eHF: infant formula manufactured from extensively hydrolysed whey protein; GI: gastrointestinal; IGSQ: Infant Gastrointestinal Symptoms Questionnaire; LSM: least square means; N: number of observations; PPS: per protocol set; SEM: standard error of mean

Table S4. Mean duration of uninterrupted nocturnal sleep (hours/night) and mean daily time to fall asleep (hours/day) from 3-day diary (PPS)

| Visit | Age at visit (days) | Uninterrupted nocturnal sleep | | | Time to fall asleep | | |
|---|---|---|---|---|---|---|---|
| | | **eHF (*n* = 149)** | **CF (*n* = 148)** | **BF (*n* = 41)** | **eHF (*n* = 149)** | **CF (*n* = 148)** | **BF (*n* = 41)** |
| 1 | 30 | 4.5 (1.5)<br>(1.3 ; 9.0) | 4.6 (1.5)<br>(1.3 ; 8.3) | 4.0 (1.8)<br>(1.6 ; 6.4) | 0.8 (0.4)<br>(0.0 ; 1.8) | 0.8 (0.4)<br>(0.1 ; 3.0) | 0.9 (0.4)<br>(0.3 ; 2.2) |
| 2 | 60 | 5.0 (1.6)<br>(1.5 ; 9.0) | 5.0 (1.6)<br>(1.8 ; 8.5) | 4.7 (1.6)<br>(1.7 ; 6.9) | 0.7 (0.3)<br>(0.1 ; 1.8) | 0.7 (0.3)<br>(0.1 ; 1.7) | 0.8 (0.3)<br>(0.2 ; 1.4) |
| 3 | 90 | 5.6 (1.8)<br>(1.5 ; 12.3) | 5.6 (1.7)<br>(1.8 ; 9.4) | 5.8 (1.8)<br>(1.8 ; 8.2) | 0.7 (0.3)<br>(0.0 ; 1.5) | 0.7 (0.3)<br>(0.2 ; 1.8) | 0.8 (0.5)<br>(0.2 ; 3.3) |
| 4 | 120 | 6.1 (1.4)<br>(1.5 ; 10.0) | 6.1 (1.6)<br>(1.8 ; 11.1) | 6.2 (1.1)<br>(3.2 ; 8.3) | 0.5 (0.2)<br>(0.2 ; 1.5) | 0.5 (0.2)<br>(0.1 ; 1.1) | 0.5 (0.2)<br>(0.1 ; 0.9) |

Values presented as mean (SD), (min ; max)

Missing values in CF for mean daily time to fall asleep: V1: *n* = 1; V2: *n* = 1

BF: breastfed group; CF: control formula; eHF: infant formula manufactured from extensively hydrolysed whey protein; *n*: number of observations; max: maximum; min: minimum; PPS: per protocol set; SD: standard deviation
